# Stool Xpert MTB/RIF as a possible diagnostic alternative to sputum in Africa: a systematic review and meta-analysis

**DOI:** 10.3389/fpubh.2023.1117709

**Published:** 2023-05-24

**Authors:** Francesco Vladimiro Segala, Roberta Papagni, Sergio Cotugno, Elda De Vita, Maria Chiara Susini, Valeria Filippi, Ottavia Tulone, Enzo Facci, Rossana Lattanzio, Claudia Marotta, Fabio Manenti, Davide Fiore Bavaro, Giuseppina De Iaco, Giovanni Putoto, Nicola Veronese, Mario Barbagallo, Annalisa Saracino, Francesco Di Gennaro

**Affiliations:** ^1^Department of Precision and Regenerative Medicine and Ionian Area, Clinic of Infectious Diseases, University of Bari, Bari, Italy; ^2^Operational Research Unit, Doctors With Africa CUAMM, Padua, Italy; ^3^Doctors With Africa CUAMM, Wolisso, Ethiopia; ^4^Geriatric Unit, Department of Internal Medicine and Geriatrics, University of Palermo, Palermo, Italy

**Keywords:** pulmonary tuberculosis, stool Xpert MTB/RIF, meta-analysis, systematic review, diagnostic microbiology

## Abstract

**Introduction:**

Worldwide, COVID-19 pandemic lead to a large fall in the number of newly reported TB cases. In sub-Saharan Africa, microbiological diagnosis of TB is generally based on smear microscopy and Xpert MTB/RIF on sputum samples, but good quality sputum samples are often difficult to obtain, leading clinicians to rely on more invasive procedures for diagnosis. Aim of this study was to investigate pooled sensitivity and specificity of Xpert MTB/RIF on stool samples compared to respiratory microbiological reference standards in African countries.

**Methods:**

Four investigators independently searched PubMed, SCOPUS, and Web of Science until 12th October 2022, then screened titles and abstracts of all potentially eligible articles. The authors applied the eligibility criteria, considered the full texts. All the studies reported the data regarding true positive (TP), true negative (TN), false positive (FP) and false negative (FN). Risk of bias and applicability concerns were assessed with the Quadas-2 tool.

**Results:**

overall, among 130 papers initially screened, we evaluated 47 works, finally including 13 papers for a total of 2,352 participants, mainly children. The mean percentage of females was 49.6%, whilst the mean percentage of patients reporting HIV was 27.7%. Pooled sensitivity for Xpert MTB/RIF assay for detecting pulmonary tuberculosis was 68.2% (95%CI: 61.1–74.7%) even if characterized by a high heterogeneity (I^2^=53.7%). Specificity was almost 100% (99%, 95%CI: 97–100%; I^2^ = 45.7%). When divided for reference standard, in the six studies using sputum and nasogastric aspirate the accuracy was optimal (AUC = 0.99, SE = 0.02), whilst in the studies using only sputum for tuberculosis detection the AUC was 0.85 (with a SE = 0.16). The most common source of bias was exclusion of enrolled patients in the analysis.

**Conclusions:**

Our study confirms that, in Africa, stool Xpert MTB/RIF may be a useful rule-in test for children above and below 5 years of age under evaluation for pulmonary tuberculosis. Sensitivity increased substantially when using both sputum and nasogastric aspirate as reference samples.

## 1. Introduction

Before the advent of COVID-19, tuberculosis was the leading cause of death from a single infectious agent, *Mycobacterium tuberculosis* (MTB). Despite being a preventable and treatable disease, it infects roughly 25% of the world population and caused at least 1.6 million deaths only in 2021, reversing a long-lasting reduction trend that started in 2000 (1). Along with an increase in TB-related deaths, the immediate consequence of the pandemic was a large fall in the number of newly reported TB cases and an estimated increase of incident cases of rifampicin-resistant TB, all indicators that represent a relevant drawback in the pursue of the 2025 End TB Milestones (2).

Since mortality of untreated TB approaches 50% and cure rates are high (3), overall disease burden is strictly dependent on diagnostic capacity. In sub-Saharan Africa, microbiological diagnosis of TB is generally based on Xpert MTB/RIF (Cepheid, USA), an automated, PCR-based assay able to detect mycobacterial DNA on respiratory samples, A newer, more sensitive version of the test has been approved by WHO in 2021, Xpert MTB/RIF Ultra, with a sensitivity approaching the one reported for culture assays (4).

In sub-Saharan Africa and other high-burden, resource-limited settings, good quality sputum samples are often difficult to obtain, leading clinicians to rely on more invasive procedures for diagnosis—such as nasogastric aspirate (5) and sputum induction, that are painful, not routinely available and require additional resources and costs such as the ones related to hospitalization and the use of suction machine and nebulizers. Besides the challenges in sample collection, MTB detection on respiratory samples in high-burden TB settings is further obstacle by extra-pulmonary tuberculosis (EPTB) (6), smear-negative pulmonary tuberculosis (PTB) and pauci-bacillary TB (7), and sputum sample collection may put healthcare workers at risk of infection due to exposure to MTB infected aerosols (8). Rapid, accurate, sputum-free diagnostics for tuberculosis are of critical need (9).

In recent years, attention has been attracted by Xpert MTB/RIF on stool samples, since mycobacteria-containing sputum may be swallowed and then be available for molecular testing. The use of Xpert MTB/RIF and Xpert MTB/RIF Ultra on stool samples has been introduced in the 2020 WHO guidelines as initial diagnostic test for children with signs and symptoms of pulmonary TB (10). However, this recommendation is based on low certainty of evidence. Also, in 2022, as part of the Global Laboratory Initiative (11) the WHO endorsed two simple, centrifuge-free methods for stool processing: the optimized sucrose flotation (OSF) method developed by the TB-Speed consortium (12), and the simple one-step (SOS) method developed by the KNCV Tuberculosis Foundation (13).

Aim of this study was to investigate pooled sensitivity and specificity of Xpert MTB/RIF on stool samples compared to respiratory microbiological reference standards in African countries.

## 2. Materials and methods

This systematic review adhered to the MOOSE guidelines (14) and PRISMA statement (15), following a predetermined but unpublished protocol.

### 2.1. Inclusion and exclusion criteria

Inclusion criteria are as follows: (i) Research highlighting the comparative assessment of the Xpert MTB/RIF or Xpert MTB/RIF Ultra assay to a reference standard, which could be either the microbiological detection of MTB (MRS, with culture, molecular or smear microscopy from either respiratory or nasogastric aspirate samples) or composite reference standard (CRS) including clinical symptoms, biochemical tests reports, radiographic results, histopathological findings, and microbiology (as defined by the authors of the individual studies), (ii) Research providing sufficient information to calculate the diagnostic performance of Xpert MTB/RIF and Xper MTB/RIF Ultra and (iii) studies conducted in African countries.

Exclusion criteria are as follows: (i) Duplicate literature studies, (ii) Research with non-human samples and animal models, (iii) Conference abstracts, lectures, commentaries, letters and case reports, (iv) Research without data (e.g., only sensitivity or specificity data), (v) performed in countries other than Africa, and (vi) publications in languages other than English.

### 2.2. Data sources and literature search strategy

Four investigators (SC, EdV, VF, MCS) independently searched PubMed, SCOPUS, and Web of Science until 12th October 2022. The search terms used in PubMed included combinations of the following keywords: (feces OR stool) AND (tuberculosis OR Mycobacterium tuberculosis OR TB OR MTB OR EPTB OR PTB) AND (Xpert Gene OR Xpert OR Xpert MTB/RI OR GeneXpert OR GeneXpert MTB/Rif). We considered the reference lists of all included articles and of previous related reviews.

### 2.3. Study selection

Following the searches as outlined above, after removal of duplicates, four independent reviewers (SC, EdV, VF, MCS) screened titles and abstracts of all potentially eligible articles. The authors applied the eligibility criteria, considered the full texts, and a final list of included articles was reached through consensus with a third senior author (NV).

### 2.4. Data extraction

Four authors were involved in data extraction in a standardized Microsoft Excel database. For each article, we extracted information about authors, year of publication, number of patients, setting, country, study design, age, percentage of females and of patients with HIV, the use of stool GeneXpert or Xpert Ultra, number of true positive, true negative, false positive and false negative results.

### 2.5. Outcomes

The primary outcomes were sensitivity, specificity, positive and negative likelihood ratios, and the area under the curve (AUC) of stool Xpert MTB/RIF and stool Xpert MTB/RIF Ultra.

### 2.6. Assessment of study quality

Based on the revised quality assessment of diagnosis, accuracy studies-2 (QUADAS-2) criteria, the included articles were evaluated as at high risk (–) or low risk (+) by four key domains: Patient selection, index test, reference standard, and flow and timing (16).

### 2.7. Data synthesis and statistical analysis

We used Meta-Disc software 5.1.4 to conduct this meta-analysis. All the studies reported the data regarding true positive (TP), true negative (TN), false positive (FP) and false negative (FN). Therefore, we were able to calculate the pooled sensitivity (TP/TP + FN), specificity (SPE) (TN/TN + FP), negative likelihood ratio (LR–), positive likelihood ratios (LR+) with their 95% confidence intervals. At the same time, we constructed the summary receiver operator characteristic (SROC) curve and calculated the area under the SROC curve based on the sensitivity and specificity of each study. Heterogeneity was estimated using the I^2^, with a value over 50% or a *p* < 0.05 as indicative of high heterogeneity. The pooled estimates were also reported by reference tool (divided in sputum vs. the association between sputum and nasogastric aspirate).

## 3. Results

**Figure 1:**
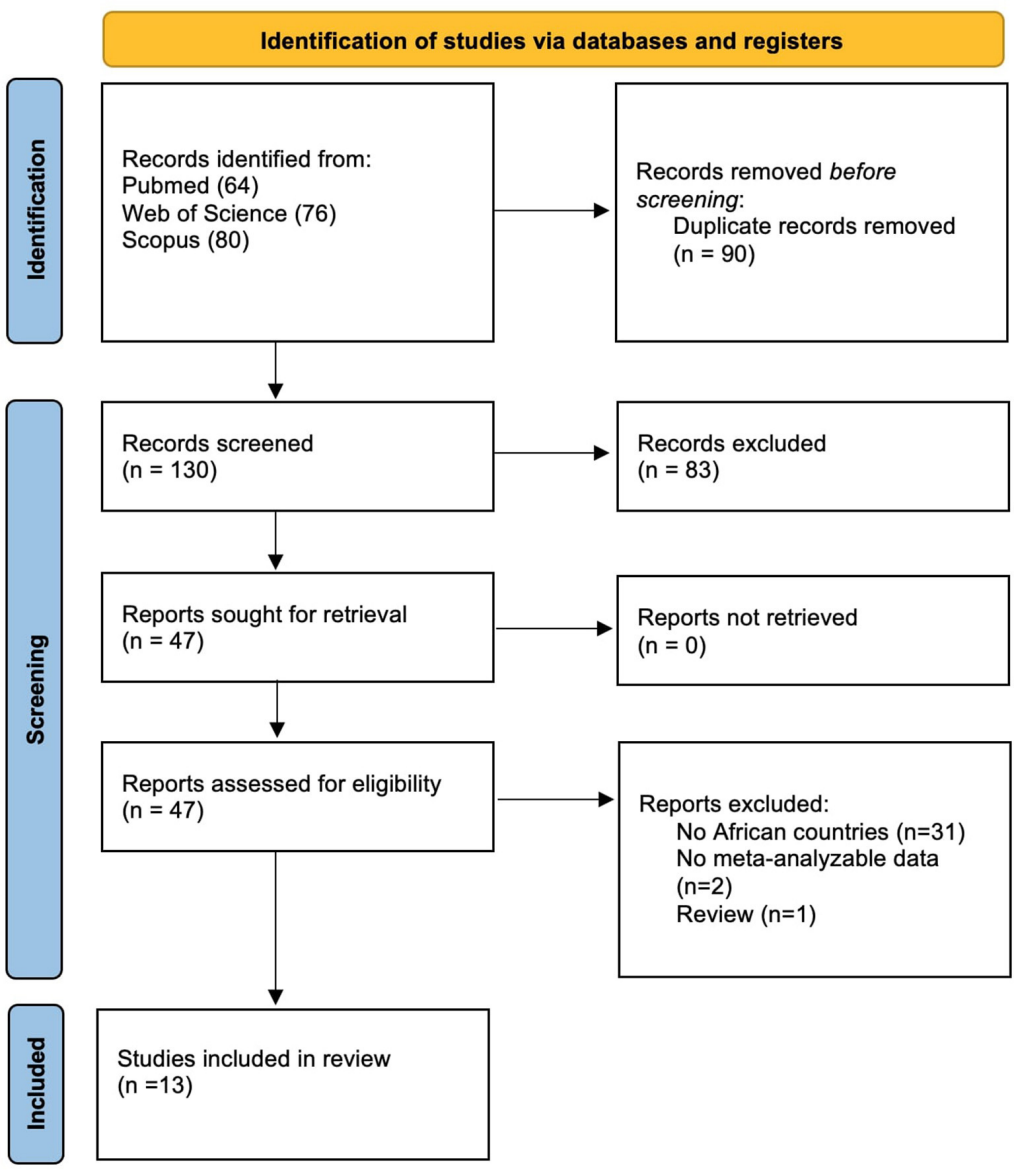
PRISMA flow-chart.

The flow-chart of this systematic review is shown in Figure 1. Overall, among 130 papers initially screened, we evaluated 47 works, finally including 13 papers.

Table 1 reported the data of the 13 works eligible for a total of 2,352 participants, mainly children. The setting most represented was the hospital (*n* = 9), followed by health center (*n* = 3) and mixed settings (*n* = 1). The mean percentage of females was 49.6%, whilst the mean percentage of patients reporting HIV was 27.7%. When considering the reference standard, the use of sputum, particularly when associated with nasogastric aspirate was the most used methodology.

**Table 1:** Descriptive characteristics of the studies included.

**Author, year**	**Country**	**Setting**	**Total sample size**	**Age at baseline; median (Q1–Q3)**	**% of females**	**%HIV**	**Stool processing method**	**Reference standard**	
								**Sample**	**Assay**
Ainan et al. (17)	Tanzania	Health Center	225	2.17 (1.16–5.19)	47.1	6.5	Homemade centrifuge free method	Sputum and nasogastric aspirate	Culture and NAAT
Banada et al. (18)	South Africa	Hospital	38	NR	55	42	Snap vortexing with stool processing buffer	Induced sputum and nasogastric aspirate	NAAT
Chipinduro et al. (19)	Zimbawe	Hospital	218	11 (9–13)	55	5.96	Stool processed using the MP Fast DNA kit for soil with a 6-minute homogenization via bead-beating disruption	Sputum	Culture, Xpert MTB/RIF and smear microscopy
DiNardo et al. (20)	Eswatini	Health Center	38	6.8 (NR)	65	32	Stool processed using the MP Fast DNA kit for soil with a 6-min homogenization via bead-beating disruption	Sputum	Culture
Dubale et al. (21)	Ethiopia	Hospital	152	3 (0.58–14)	51.3	NR	Single step, centrifuge-free protocol adapted from KNCV TB foundation (13)	Sputum and nasogastric aspirate	Culture, Xpert MTB/RIF and smear microscopy
Lacourse et al. (22)	Kenya	Hospital	164	2 (13–58)	43.4	100	Sedimentation based method with centrifugation	Sputum and nasogastric aspirate	Culture and Xpert MTB/RIF
Moussa et al. (23)	Egypt	Hospital	115	NR	40.33	0	Sedimentation based method with centrifugation	Sputum	Culture
Nicol et al. (24)	South Africa	Health Center	115	2.58 (19–57)	NR	14.8	Supernatant-based method with centrifugation	Induced sputum and nasogastric aspirate	Culture
Orikiriza et al. (25)	Uganda	Hospital	392	NR	45.5	31.2	Sedimentation based method with centrifugation	Sputum	Culture
Orikiriza et al. (26)	Uganda	Hospital	219	1.36 (9.7–29.7)	48.9	32	Sedimentation based method with centrifugation	Any sample (excluding stool)	Culture and Xpert MTB/RIF
De Haas et al. (13)	Ethiopia	Hospital	123	NR	NR	NR	Single step, centrifuge-free protocol adapted from KNCV TB foundation (13)	Sputum and nasogastric aspirate	Culture and Xpert MTB/RIF Ultra
Song et al. (27)	Kenya	Mixed	294	2 (1–3.6)	50.3	23	Not described	Sputum	Culture and Xpert MTB/RIF
Walters et al. (28)	South Africa	Hospital	259	1.29 (0.88–2.4)	43.6	12.5	Sedimentation based method with centrifugation	Sputum	Culture and Xpert MTB/RIF

NR, not reported.

**Figure 2:**
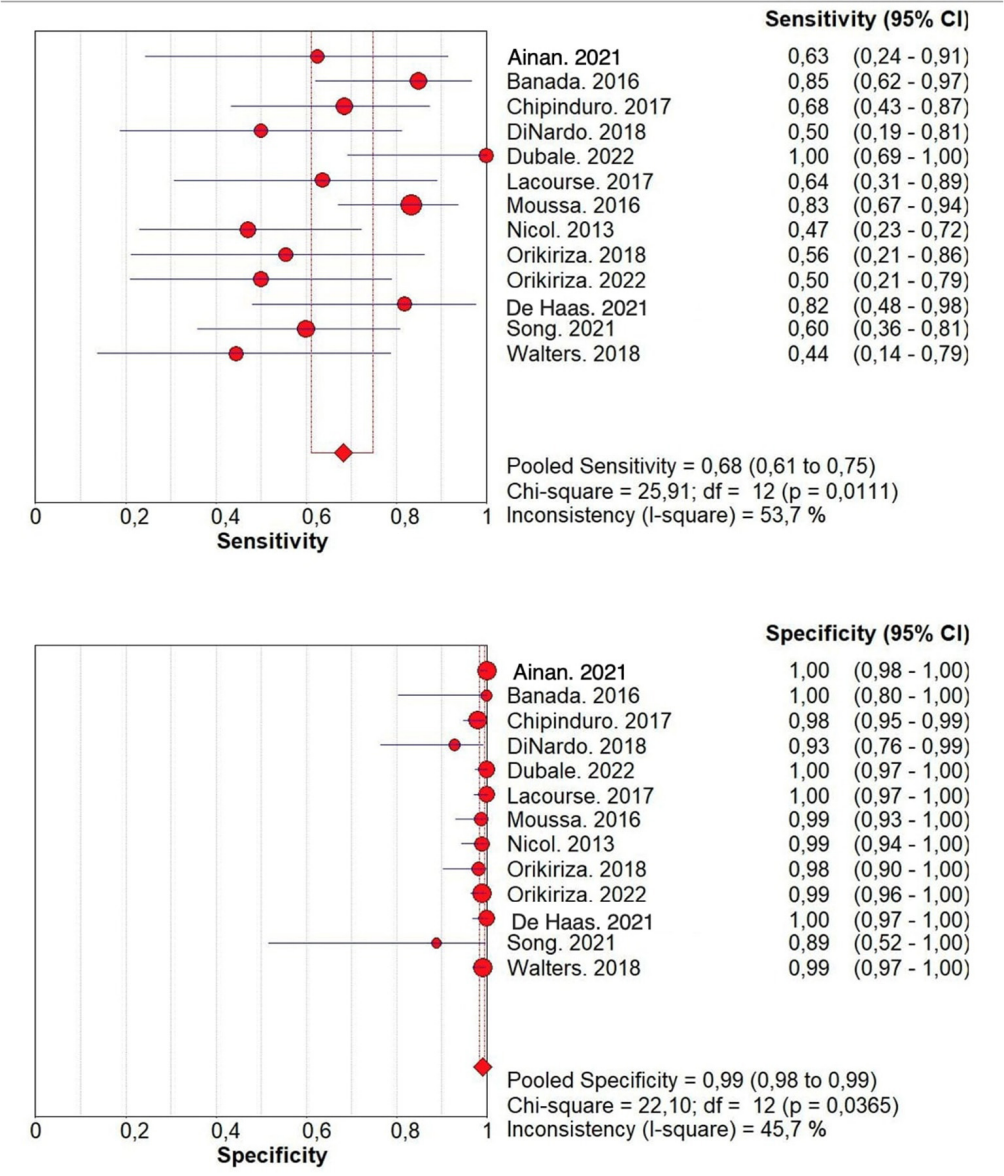
Forest plots of sensitivity and specificity of the Xpert MTB/RIF Ultra assay for tuberculosis detection. Power of the single studies is indicated by dot size, while horizontal lines indicate emerging from the box indicate the magnitude of the confidence interval. Dot size is proportional to the studies' sample size. and that the lozenge and box represents the pooled numbers with 95% CI error margins.

**Figure 3:**
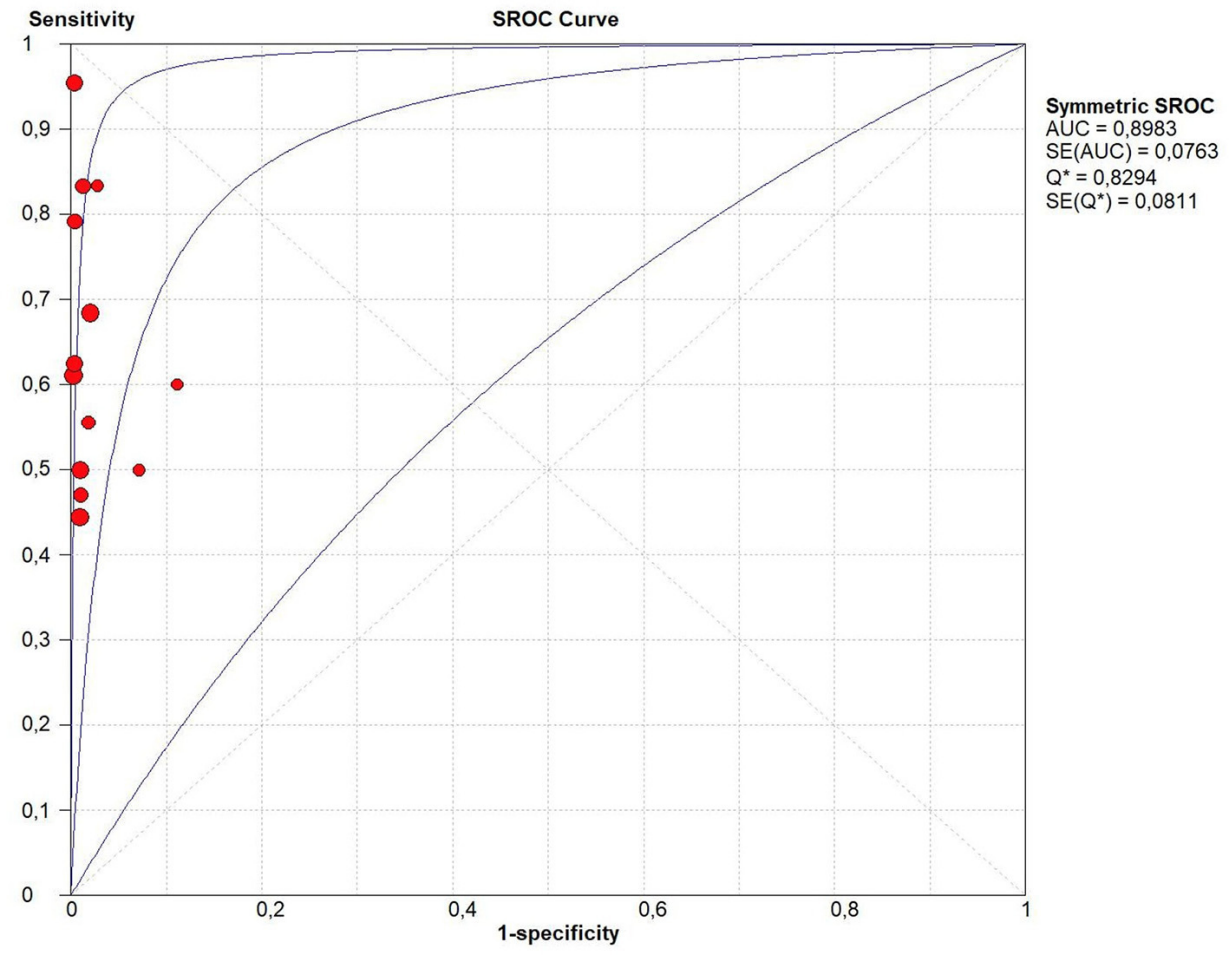
Summary receiver operating characteristic (SROC) curve of the diagnostic accuracy of Xpert MTB/RIF Ultra assay for tuberculosis detection. In this figure, the blue lines represent the AUC (central line) with its 95% CI (external lines) calculated with a meta-analytic approach, while red dots represent the sensitivity and specificity data for each study.

Considering all the 13 studies together, the pooled sensitivity for stool Xpert MTB/RIF assay for detecting tuberculosis was moderate (68.2%, 95%CI: 61.1–74.7%) even if characterized by a high heterogeneity (I^2^ = 53.7%) (Figure 2). In fact, the sensitivity of the studies included ranged from 44% to 100%. On the contrary the specificity of stool Xpert assay was almost 100% (99%, 95%CI: 97–100%; I^2^=45.7%) (Table 2, Figure 2). Almost all the studies reported a specificity higher than 95% in diagnosing tuberculosis, as shown in Figure 2. Therefore, the LR+ was optimal (38.581; 95%CI: 20.994–70.900) as well as the LR- (0.383; 95%CI: 0.295- 0.497) (Table 2). These data led to an AUC = 0.8983 with a standard error (SE) of 0.0763, even if, as shown in Figure 3, only four studies had an AUC over 0.80.

**Table 2:** Performance of Xpert MTB/RIF Ultra on stool sample from patients with pulmonary tuberculosis compared to reference standard type.

	**Number of studies**	**Pooled sensitivity (95% CI)**	**Pooled specificity (95% CI)**	**Positive Likelihood ratio (95% CI)**	**Negative likelihood ratio (95% CI)**
All sample	13	0.682 (0.611–0.747)	0.991 (0.985–0.995)	38.581 (20.994–70.900)	0.383 (0.295–0.497)
Sputum and nasogastric aspirate	6	0.727 (0.614–0.823)	0.999 (0.992–1.000)	105.78 (36.708–304.802)	0.317 (0.189–0.533)
Only sputum	6	0.670 (0.570–0.759)	0.981 (0.967–0.991)	22.884 (10.407–50.321)	0.397 (0.274–0.574)

CI, Confidence Intevals.

Stratifying the analysis for the reference standard, the six studies using both sputum and nasogastric aspirate showed higher sensitivity, a similar specificity, and a higher LR+ than studies using only sputum for tuberculosis detection, as shown in Table 1. In the six studies using sputum and nasogastric aspirate the accuracy was optimal (AUC = 0.99, SE = 0.02), whilst in the studies using only sputum for tuberculosis detection the AUC was 0.85 (with a SE = 0.16). In our systematic review, despite the initial protocol included both composite and microbiological reference standards, we found only one study evaluating diagnostic accuracy of stool Xpert with both CRS and MRS (26). In this case, interestingly, sensitivity dropped from 50% against MRS to 11.4% against CRS. However, for this study, we included in the meta-analysis only diagnostic accuracy data obtained against MRS.

**Figure 4:**
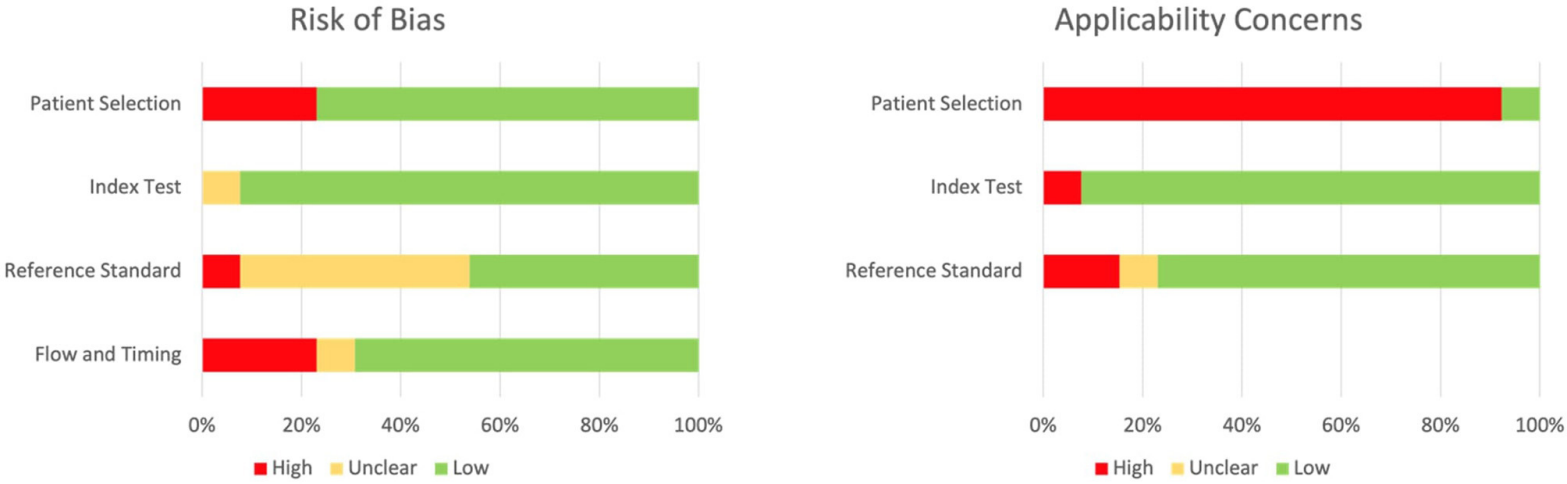
QUADAS-2 risk of bias and applicability concerns graph.

The quality of the included studies, as assessed by the QUADAS-2, is reported on Figure 4; the most common source of bias was exclusion of enrolled patients in the analysis (Figure 4). On the other side, the most common concern in terms of applicability was due to the fact that our systematic review aimed to explore the diagnostic accuracy of stool Xpert in the general population, while most of the included studies recruited only pediatric patients. A detailed description of risk of bias and applicability concerns is provided in Supplementary Table 1.

## 4. Discussion

In this systematic review and meta-analysis, we investigated diagnostic accuracy of stool Xpert MTB/RIF in African settings. In our study, pooled sensitivity and specificity were, respectively, 68% (95%CI 61–75%) and 99% (95%CI 98–99%) for the diagnosis of people with presumptive pulmonary TB. Our results are consistent with the ones reported by other meta-analysis conducted on children living in both African and non-African settings (29–31). Moreover, also consistently with other studies, diagnostic accuracy reported here on stool samples is comparable to the performances of the same test on respiratory samples (32). Of note, this is the first meta-analysis including only patients living in African countries.

In low-resource settings, patients evaluated for TB often experience diagnostic delays due to several factors, such as economic constrains, lack of awareness on the importance of timely diagnosis and poor availability of diagnostic tools in primary healthcare facilities (9). This scenario was further challenged by the COVID-19 pandemic, that reversed the progresses made during the last decades and lead, worldwide, to a large drop in the reported number of newly diagnosed TB (1). This is relevant, since every loss in TB diagnostic capacity inevitably leads to an increase in the number of untreated TB and TB deaths. Despite their requirements in costs and infrastructure—which limits the availability to the settings with adequate transportation systems and funding—molecular tests such as Xpert MTB/RIF and Xpert MTB/RIF Ultra on stool sample may provide added value in TB diagnostic workflow in high burden settings. Also, another limit of rapid molecular tests is that they are sputum dependent, since population at high risk of developing TB [such as people living with HIV (33) and children (34)] is often unable to expectorate. Among children, sputum unavailability is generally replaced by nasogastric aspirate, which is invasive and poorly tolerated. In our systematic review, 7 studies out of 13 reported a median age below 5 years, accounting for 66.7% (*n* = 1,571) of the pooled population, that is the age category in which stool Xpert is expected to have the greatest clinical utility. The importance of implementing rapid, accurate, non-invasive, sputum-free assays for detection of MTB has been recognized by the WHO as high priority target for the development of new tuberculosis diagnostics in 2014 (35).

Consistently with other meta-analysis (29–31), we recorded a substantial between-study heterogeneity, especially in sensitivity, which ranged from 44% to 100%. This was likely a consequence of the differences in reported HIV-prevalence and in terms of used reference test. Furthermore, heterogeneity might also have been affected by the stool processing protocols used, since the majority of included articles reported non-standardized sample processing methods. In this study we found that, despite WHO endorsement of SOS and OSF methods, implementation of stool Xpert processing strategies in sub-Saharan Africa is still lacking standardization. This is relevant, since many in-house, not-standardized methods require laboratory expertise dedicated equipment which, in some settings, may discourage implementation of PCR-based diagnostics on stool samples. For future, perspective research, we emphasize the importance to adopt and report a standardized protocol for sample preparation.

A strength of this study is that diagnostic accuracy was evaluated using, in all articles, a microbiological (and non-clinical) reference standard, represented by both culture and Xpert (8/13), culture (12/13), or Xpert alone (1/13). In fact, when the reference standard used to evaluate diagnostic accuracy of stool Xpert was both sputum and nasogastric aspirate, pooled sensitivity increased to 72% and AUC was as high as 0.99.

This study has some limitations. First, data did not allow us to perform meta-regression analyses to investigate the reasons of recorded heterogeneity. Second, we could not evaluate the accuracy of stool Xpert on adults or other age groups, since we found no studies addressing this population in African countries. Third, we found only one study investigating diagnostic performances of stool Xpert Ultra (13), which contributed for 5% of the total population and reported a sensitivity of 81%. For the purposes of this study, Xpert Ultra has been included in the analysis but we recognize that it may have contributed to increase heterogeneity. Hence, future research should focus on investigating diagnostic accuracy and cost-effectiveness of Xpert Ultra on stool samples in sub-Saharan settings. Also, in the upcoming years, research should address the sensitivity advantage of this test on adults and when used in combination with other currently used assays.

## 5. Conclusions

Our study confirms that, in Africa, stool Xpert MTB/RIF may be a useful rule-in test for patients under evaluation for pulmonary tuberculosis. Sensitivity increased substantially when using both sputum and nasogastric aspirate as reference samples. Further studies are needed to explore stool Xpert MTB/RIF Ultra and both Xpert MTB/RIF and Xpert Ultra diagnostic performance in the adult population.

## Data availability statement

The original contributions presented in the study are included in the article/Supplementary material, further inquiries can be directed to the corresponding author.

## Author contributions

FD, CM, and NV contributed to conception and design of the study. RP, SC, ED, MS, VF, OT, EF, RL, FM, DB, and GD collected the data. FV organized the database and wrote the first draft of the manuscript. NV performed the statistical analysis. GP, NV, FD, and MB wrote sections of the manuscript. All authors contributed to manuscript revision, read, and approved the submitted version.
