# Supplementary material for: Stool Xpert MTB/RIF as a possible diagnostic alternative to sputum in Africa: a systematic review and meta-analysis
Source: Front Public Health. 2023 May 24;11:1117709. doi: 10.3389/fpubh.2023.1117709 (PMC10244509; doi:10.3389/fpubh.2023.1117709)
Supplement: Supplementary file 1 [file Data_Sheet_1.PDF]

	Risk of bias														
	Patient selection				studio testing			Reference standard			Flow and timing				
	Was a consecutive or random sample of patients enrolled?	Was a case-control study design avoided?	Did the study avoid inappropriate patient exclusions?	**Could patient selection be a source of bias?**	Were the results of the test under study interpreted without knowing the results of the reference standard?	Was the threshold value, if any, used predefined?	**Could the administration and interpretation of the test in the study be a source of bias?**	Is the reference standard adequate to correctly classify the target population?	The results of the reference standard were interpreted without knowing the results of the test under study	**Could the administration and interpretation of the reference standard be a source of bias?**	Is the time interval between performing the study test and the reference standard adequate?	Did all patients receive the same reference standard?	Were all enrolled patients included in the analysis?	**Could patient flow be a source of bias?**
Ainan, 2021	YES	YES	YES	**LOW RISK.**	NOT CLEAR	Yes	NOT CLEAR	NOT CLEAR	NOT CLEAR	**NOT CLEAR**	YES	YES	NO	**LOW RISK.**
Banada, 2016	YES	NO	NOT CLEAR	**HIGH RISK.**	NO	YES	LOW RISK.	NO	NO		NOT CLEAR	YES	YES	**LOW RISK.**
Chipinduro, 2017	YES	YES	NO	**LOW RISK.**	YES	YES	LOW RISK.	NO	YES	**LOW RISK.**	YES	YES	NO	**LOW RISK.**
DiNardo, 2018	YES	YES	YES	**LOW RISK.**	YES	YES	LOW RISK.	YES	YES	**LOW RISK.**	NO	YES	YES	**LOW RISK.**
Dubale, 2022	YES	YES	YES	**LOW RISK.**	NOT CLEAR	YES	LOW RISK.	NOT CLEAR	NOT CLEAR		YES	YES	NO	**LOW RISK.**

Lacourse, 2017	NOT CLEAR	YES	NO	**HIGH RISK.**	NOT CLEAR	YES	LOW RISK.	NOT CLEAR	NOT CLEAR	**NOT CLEAR**	YES	YES	NO	**LOW RISK.**
Moussa, 2016	YES	YES	NOT CLEAR	**LOW RISK.**	YES	YES	LOW RISK.	YES	NOT CLEAR		YES	YES	NO	**LOW RISK.**
Nicol, 2013	YES	YES	YES	**LOW RISK.**	NOT CLEAR	YES	LOW RISK.	NOT CLEAR	NOT CLEAR	**NOT CLEAR**	YES	YES	YES	**LOW RISK.**
Orikiriza, 2018	YES	YES	YES	**LOW RISK.**	NOT CLEAR	YES	LOW RISK.	NOT CLEAR	NOT CLEAR	**NOT CLEAR**	YES	NO	NO	**HIGH RISK.**
Orikiriza, 2022	NOT CLEAR	YES	NO	**HIGH RISK.**	NOT CLEAR	YES	LOW RISK.	YES	NOT CLEAR	**LOW RICH.**	YES	NO	NO	**HIGH RISK.**
De Haas, 2021	YES	YES	YES	**LOW RISK.**	NOT CLEAR	YES	LOW RISK.	NOT CLEAR	NOT CLEAR	**NOT CLEAR**	NOT CLEAR	YES	NO	**LOW RISK.**
Song, 2021	YES	YES	YES	**LOW RISK.**	YES	YES	LOW RISK.	NOT CLEAR	YES	**LOW RICH.**	YES	NO	NOT CLEAR	**NOT CLEAR**
Walters, 2018	NOT CLEAR	YES	YES	**LOW RISK.**	NOT CLEAR	YES	LOW RISK.	YES	NOT CLEAR	**LOW RICH.**	NOT CLEAR	NO	NO	**HIGH RISK.**

	Applicability issues		
	**Patient selection**	**In-studio testing**	**Reference standard**
	**Could the patients included and the enrollment setting not match the RS question?**	**Could the study test, its performance or interpretation differ from the RS question?**	**Could the target condition defined by the reference standard not match the RS question?**
Ainan, 2021	**HIGH RISK.**	**LOW RISK.**	**LOW RISK.**
Banada, 2016	**HIGH RISK.**	**LOW RISK.**	**NOT CLEAR**
Chipinduro, 2017	**HIGH RISK.**	**LOW RISK.**	**LOW RISK.**
DiNardo, 2018	**LOW RISK.**	**HIGH RISK.**	**LOW RISK.**
Dubale, 2022	**HIGH RISK.**	**LOW RISK.**	**LOW RISK.**

Lacourse, 2017	**HIGH RISK.**	**LOW RISK.**	**LOW RISK.**
Moussa, 2016	**HIGH RISK.**	**LOW RISK.**	**LOW RISK.**
Nicol, 2013	**HIGH RISK.**	**LOW RISK.**	**LOW RISK.**
Orikiriza, 2018	**HIGH RISK.**	**LOW RISK.**	**LOW RISK.**
Orikiriza, 2022	**HIGH RISK.**	**LOW RISK.**	**HIGH RISK.**
De Haas, 2021	**HIGH RISK.**	**LOW RISK.**	**LOW RISK.**
Song, 2021	**HIGH RISK.**	**LOW RISK.**	**HIGH RISK.**
Walters, 2018	**HIGH RISK.**	**LOW RISK.**	**LOW RISK.**
