# Supplementary material for: Stool Xpert MTB/RIF as a possible diagnostic alternative to sputum in Africa: a systematic review and meta-analysis
Source: Front Public Health. 2023 May 24;11:1117709. doi: 10.3389/fpubh.2023.1117709 (PMC10244509; doi:10.3389/fpubh.2023.1117709)
Supplement: Supplementary file 2 [file Data_Sheet_2.docx]

**GENEXPERT OF STOOL SAMPLES IN AFRICA: A META-ANALYSIS**

**Protocol synopsis**

**Author: Francesco Vladimiro Segala**

**Version: 1.0**

**SYNOPSIS**

| Background and aim | Worldwide, COVID-19 pandemic lead to a large fall in the number of newly reported TB cases. In Africa, microbiological diagnosis of TB is generally based on smear microscopy and Xpert MTB/RIF on sputum samples, but good quality sputum samples are often difficult to obtain, leading clinicians to rely on more invasive procedures for diagnosis. Aim of this study was to investigate pooled sensitivity and specificity of Xpert MTB/RIF on stool samples compared to respiratory microbiological reference standards in African countries. |
| --- | --- |
| Study design | Systematic review and meta-analysis |
| Inclusion criteria | - Research highlighting the comparative assessment of the Xpert MTB/RIF and Xpert MTB/RIF Ultra on stool samples to a reference standard: Mtb culture/GeneXpert or combined reference standard (CRS) including clinical symptoms, biochemical tests reports, radiographic results, histopathological findings, and smear microscopy, as defined by the authors of the individual studies. - Research providing sufficient information to calculate the diagnostic performance of stool Xpert MTB/RIF and stool Xpert MTB/RIF Ultra using the standard indicators: true positives, false positives, true negatives, false negatives. |
| Exclusion criteria | - Duplicate literature studies - Research with non-human samples and animal models - Conference abstracts, lectures, commentaries, letters and case reports - Research with no meta-analyzable data (e.g., only sensitivity or specificity data) - Performed in continents other than Africa. - Publications in languages other than English. |
| Search strategy | The search terms used in PubMed will include combinations of the following keywords: (feces OR stool) AND (tuberculosis OR Mycobacterium tuberculosis OR TB OR MTB OR EPTB OR PTB) AND (Xpert Gene OR Xpert OR Xpert MTB/RI OR GeneXpert OR GeneXpert MTB/Rif). Reference lists of all included articles and of previous related reviews will be considered. |
| Study selection and data extraction | Following the searches as outlined above, after removal of duplicates, at least 3 independent reviewers will screen titles and abstracts of all potentially eligible articles. The reviewers will apply the eligibility criteria on the full texts, and a final list of included articles will be obtained through consensus with one or more senior authors. |
| Data extraction | At least three authors will be involved in data extraction in a standardized Microsoft Excel database. For each article, we will extract information about authors, year of publication, number of patients, setting, country, study design, age, percentage of females and of patients with HIV, the use of stool GeneXpert or Xpert Ultra, number of true positive, true negative, false positive and false negative results. |
| Outcomes | The primary outcomes will be sensitivity, specificity, positive and negative likelihood ratios, and the area under the curve (AUC) of the presence of tuberculosis in patients living in Africa. |
| Assessment of study quality | Based on the revised quality assessment of diagnosis, accuracy studies-2 (QUADAS-2) criteria, the included articles were evaluated as at high risk or low risk by four key domains: Patient selection, index test, reference standard, and flow and timing. |
| Data synthesis and statistical analysis | All studies reporting the data regarding true positive (TP), true negative (TN), false positive (FP) and false negative (FN) will be used to calculate pooled sensitivity (TP/TP + FN), specificity (SPE) (TN/TN + FP), negative likelihood ratio (LR−), positive likelihood ratios (LR+) with their 95% confidence intervals. We will also construct the summary receiver operator characteristic (SROC) curve and calculated the area under the SROC curve based on the sensitivity and specificity of each study. Heterogeneity will be estimated using the I2, with a value over 50% or a p-value <0.05 as indicative of high heterogeneity. The pooled estimates will also be reported by reference tool. |
